# Trends in physical fitness among polish children and adolescents

**DOI:** 10.3389/fpubh.2025.1576822

**Published:** 2025-11-26

**Authors:** Joanna Baj-Korpak, Kamil Zaworski, Marek Wochna, Sebastian Chmara, Marian J. Stelmach

**Affiliations:** 1 Department of Health Sciences, John Paul II University in Biala Podlaska, Biala Podlaska, Poland; 2 Polish Athletics Association, Warsaw, Poland

**Keywords:** physical fitness, trends, OSF test, Body Mass Index, Ponderal Index, children, adolescents, gender

## Abstract

**Background:**

Human health, with physical fitness as one of its key indicators, is characterized by a dynamic nature. Therefore, monitoring motor abilities and the factors that influence them becomes particularly important. Recent studies on physical fitness trends among children and adolescents indicate a declining tendency, with a noticeable decrease in physical performance. Maintaining an optimal level of physical fitness in young individuals requires support not only from parents. Hence, health promotion policies should focus on fostering health-related physical fitness from early childhood. The aim of the current study was to determine physical fitness trends among Polish children and adolescents participating in the nationwide program “Athletics for All!” (AFA). An attempt was made to answer the question of whether the implementation of programs promoting physical activity among youth (as exemplified by the AFA program) has an impact on improving physical fitness and body build indicators as its markers.

**Methods:**

Physical fitness was assessed in a group of 54,049 young individuals, including 31,789 girls and 22,260 boys, over eight consecutive years of the AFA program’s implementation. The assessment was conducted using the OSF test, developed specifically for the AFA program, which includes a 3×10 m shuttle run, standing broad jump, 1 kg medicine ball throw, and a 4-min run. Statistical analyses were performed separately for each gender, assuming a significance level of alpha = 0.05. It was verified whether there were significant differences in the means between the groups distinguished based on the year of the study.

**Results:**

The study results indicate that the implementation of the AFA program promoting physical activity among Polish youth has a positive impact on the improvement of physical fitness and body build parameters. Statistically significant differences were found in the mean values of measurements taken over the course of eight consecutive years. The results of multiple comparisons between the mean values of all the analyzed variables in different years revealed significant differences in most of the comparisons.

**Conclusion:**

The level of physical fitness among participants of the AFA program remained relatively stable over nearly a decade, which, in an era dominated by a sedentary lifestyle among modern youth, confirms the validity of implementing physical activity promotion programs (including the AFA program).

## 1 Introduction

Physical fitness (PF) is defined as “the ability to carry out daily tasks with vigor and alertness, without undue fatigue, and with ample energy to enjoy leisure-time pursuits and to meet unforeseen emergencies” (1). Physical fitness is a set of attributes that reflect an individual’s ability to engage in physical activity and typically includes measurements of cardiorespiratory fitness, muscle strength and power, flexibility, and body composition (2).

A high level of physical fitness is positively associated with health (3)—it provides unique information related to the potentially healthy growth and development of children and adolescents (2). It is considered an important indicator of current (4) and future (5) health, regardless of the physical activity undertaken (6). As emphasized by (7), physical fitness is an important health resource, representing a comprehensive measure of the body’s ability to engage in physical activity. Moreover, researchers point out the transfer of behaviors into adulthood, where active children are more likely to become more active (healthy) adults (8).

PF can be described using two models. The first one is related to skills and is primarily used to assess physical fitness in athletes. The other one is associated with health. Health-related PF includes muscle strength, speed/agility, cardiorespiratory fitness, and body composition (4).

In children and adolescents, a high level of PF is associated with health benefits, such as stronger bones, better quality of life, higher self-esteem, improved cognitive performance, and a lower incidence of cardiovascular disease risk factors (6, 9, 10). Conversely, low muscle strength and cardiorespiratory fitness during adolescence are linked to risk factors for the leading causes of death in adulthood (11, 12). Cardiorespiratory fitness is positively correlated with improved health in children and adolescents (13, 14). However, research indicates that the level of cardiorespiratory fitness in children is declining (14, 15). It is important to emphasize that this ability is more strongly associated with health outcomes compared to measurements of muscle strength or flexibility (3, 16). Furthermore, large cohort studies have shown a link between low cardiorespiratory fitness in late adolescence and early mortality (11, 17). Robinson et al. (17) report that endurance correlates with cardiovascular-metabolic risk in children.

Robust and consistent evidence confirms that physical fitness is a strong marker of health in children and adolescents (6, 18). Among the various components of physical fitness, cardiorespiratory fitness (endurance) and muscular strength (strength) have shown the strongest associations with health and are therefore considered the primary components of health-related fitness (10, 19).

In recent decades, a clear secular trend toward a higher Body Mass Index and poorer physical fitness levels has been observed among Polish children (20, 21). Therefore, health promotion policies should focus on maintaining proper health-related physical fitness levels from early childhood (4).

In addition to its health implications, physical fitness is an important determinant of success in many sports, including athletics (22).

The physical fitness of school-aged children and adolescents can be considered from two perspectives: current status and the dynamics of change. As previously mentioned, physical fitness (PF) serves as a marker of both present and future health. Motor abilities exhibit a potentially bidirectional relationship with physical activity and health-related fitness (23). For this reason, there is a need for simple yet reliable measurement methods (5). It is also essential to adopt a diversified approach to physical activity, taking into account gender, age, and fitness levels (23, 24). Normative physical fitness values, which place individuals and groups within percentiles and categories, can be useful for interpreting individual fitness test results, determining how an individual’s performance compares to the general population, and identifying athletic talent (25). Beyond assessing the overall PF of children and adolescents, tracking trends is particularly important to identify specific contexts in which physical fitness may decline (26). Such actions should be aimed at intervention. It is evident that, despite natural tendencies, children have become increasingly less physically active in recent decades (8). Consequently, physical activity and fitness have become key areas of research in relation to the unsatisfactorily high rates of overweight and obesity among younger generations (26). Numerous studies have found that the Ponderal Index (PI) is more accurate and stable than the Body Mass Index (BMI) in indicating levels of overweight, obesity (27–29), and metabolic syndromes (30) in children and adolescents. PI is a valuable tool for analyzing and interpreting adolescent physical fitness, as it accounts for significant differences in body mass status between study participants (31).

The aim of the current study was to determine trends in the physical fitness of Polish children and adolescents participating in the “Athletics for All!” program. This publication attempts to answer the question of whether the implementation of programs promoting physical activity among youth (as exemplified by the AFA program) contributes to the improvement in physical fitness and body build indicators as its markers.

## 2 Materials and methods

### 2.1 Participants

The study on the physical fitness of Polish youth was conducted as part of the nationwide AFA program, implemented between 2015 and 2022. Over the eight years of the program’s operation, physical fitness measurements were taken from a group of 54,049 young individuals, including 31,789 girls and 22,260 boys. Table 1 presents the general characteristics of the studied population, taking into account the year of the study, as well as age and gender.

**Table 1:** Characteristics of the study group (*N* = 54,049).

Age (years)	Gender	N/%	Year of study implementation								Total
			2015	2016	2017	2018	2019	2020	2021	2022	
8	F	N	12	53	0	54	75	50	85	71	400
		%	3.0	13.3	0.0	13.5	18.8	12.5	21.3	17.8	100.0
	M	N	11	40	0	47	65	70	48	66	347
		%	3.2	11.5	0.0	13.5	18.7	20.2	13.8	19.0	100.0
9	F	N	161	214	20	383	183	147	111	113	1,332
		%	12.1	16.1	1.5	28.8	13.7	11.0	8.3	8.5	100.0
	M	N	114	162	6	271	152	124	119	66	1,014
		%	11.2	16.0	0.6	26.7	15.0	12.2	11.7	6.5	100.0
10	F	N	525	986	40	875	871	585	381	226	4,489
		%	11.7	22.0	0.9	19.5	19.4	13.0	8.5	5.0	100.0
	M	N	525	713	28	615	588	425	251	154	3,299
		%	15.9	21.6	0.8	18.6	17.8	12.9	7.6	4.7	100.0
11	F	N	836	1,352	78	1,320	1,239	956	800	527	7,108
		%	11.8	19.0	1.1	18.6	17.4	13.4	11.3	7.4	100.0
	M	N	579	1,099	74	945	933	611	551	334	5,126
		%	11.3	21.4	1.4	18.4	18.2	11.9	10.7	6.5	100.0
12	F	N	805	1,467	259	1,377	1,257	1,132	914	842	8,053
		%	10.0	18.2	3.2	17.1	15.6	14.1	11.3	10.5	100.0
	M	N	527	1,012	199	832	881	788	532	557	5,328
		%	9.9	19.0	3.7	15.6	16.5	14.8	10.0	10.5	100.0
13	F	N	334	654	280	694	728	719	856	737	5,002
		%	6.7	13.1	5.6	13.9	14.6	14.4	17.1	14.7	100.0
	M	N	313	505	167	445	432	433	581	402	3,278
		%	9.5	15.4	5.1	13.6	13.2	13.2	17.7	12.3	100.0
14	F	N	84	534	36	482	333	386	588	569	3,012
		%	2.8	17.7	1.2	16.0	11.1	12.8	19.5	18.9	100.0
	M	N	55	523	16	317	170	200	427	397	2,105
		%	2.6	24.8	0.8	15.1	8.1	9.5	20.3	18.9	100.0
15	F	N	31	162	25	449	262	330	328	304	1891
		%	1.6	8.6	1.3	23.7	13.9	17.5	17.3	16.1	100.0
	M	N	25	147	0	361	192	207	208	239	1,379
		%	1.8	10.7	0.0	26.2	13.9	15.0	15.1	17.3	100.0
16	F	N	1	2	0	106	63	212	26	9	419
		%	0.2	0.5	0.0	25.3	15.0	50.6	6.2	2.1	100.0
	M	N	0	1	1	69	61	153	12	17	314
		%	0.0	0.3	0.3	22.0	19.4	48.7	3.8	5.4	100.0
17	F	N	0	1	0	2	26	46	4	4	83
		%	0.0	1.2	0.0	2.4	31.3	55.4	4.8	4.8	100.0
	M	N	0	0	0	2	24	41	3	0	70
		%	0.0	0.0	0.0	2.9	34.3	58.6	4.3	0.0	100.0
Total	F	N	2,789	5,425	738	5,742	5,037	4,563	4,093	3,402	31,789
		%	8.8	17.1	2.3	18.1	15.8	14.4	12.9	10.7	100.0
	M	N	2,149	4,202	491	3,904	3,498	3,052	2,732	2,232	22,260
		%	9.7	18.9	2.2	17.5	15.7	13.7	12.3	10.0	100.0
Σ		N	4,938	9,627	1,229	9,646	8,535	7,615	6,825	5,634	54,049
		%	9.1	17.8	2.3	17.8	15.8	14.1	12.6	10.4	100.0

The inclusion criteria for the study required participation in the AFA program, confirmed by written consent from a parent or legal guardian (which also served as a declaration of no contraindications for participation in physical activities and fitness tests) and an age range of 8 to 17 years. Considering that children aged 8 and 9 are in early primary education (following a different curriculum than students in grades IV-VIII of the Polish primary school system), an additional criterion for including results in further analyses was a minimum of two years of participation in the AFA program.

Due to the lack of a reference group (for 8- and 17-year-olds), insufficient program participation time (for 9-year-olds), and the small sample sizes of certain age groups (16- and 17-year-olds), the following participants were excluded from the analysis: 747 (400 F and 347 M) 8-year-olds, 2,346 (1,332 F and 1,014 M) 9-year-olds, 733 (419 F and 314 M) 16-year-olds, and 153 (83 F and 70 M) 17-year-olds. Ultimately, the analysis included 50,070 participants of the AFA program (Supplementary material).

The program was created out of the need to popularize athletics as an introductory sport for children and youth. Thanks to this initiative, athletics is presented as an attractive sport that brings joy to younger children and provides adolescents with satisfaction through physical activity, peer competition, and the acquisition of new skills. Athletics for All! has been implemented since mid-2014. Currently, the program includes over 600 training groups across Poland.

The “Athletics for All!” program was carried out in three stages. The first stage included students from grades I-IV of primary schools (7–10 years old). For children in the younger primary school grades, athletics-based physical activities were offered. Coaches working with the youngest participants of the AFA program conducted sessions twice a week, during which children began their journey with athletics in the form of play. The second stage involved students from grades V-VIII of primary schools (11–14 years old). Participants from this age group had access to regular sports sessions and took part in a series of athletic tests and competitions. This stage also focused on the selection and recruitment of the youngest athletes, as well as the identification of athletic talents. The sessions were held three times a week and lasted for one and a half hours. The most talented children had the opportunity to continue their careers in the so-called Centers of Oriented Training (COTs) – the third stage (for youth up to 17 years of age). Training in these groups is more advanced, with young athletes participating in sports competitions and training camps, and regularly undergoing physical fitness tests.^1^

The main objectives of the AFA program are to promote and popularize athletics, create an attractive offer for organizing sports activities for children and youth from diverse backgrounds, develop a coherent training process model in line with the guidelines of the world and European athletics federations, establish a nationwide system for diagnosis, selection, recruitment, and talent identification in youth training, as well as build a career development pathway in athletics that would form the foundation of a new training structure for children and youth in Poland.

### 2.2 Methods

Physical fitness measurements were conducted using the OSF test. This tool was developed specifically for the AFA program. The authors of this publication validated and standardized this test (32). The OSF test assesses four key motor abilities: speed, power, strength, and endurance. These are fundamental motor skills, primarily from the perspective of sports training (athletic talent identification), but also from the perspective of health-related training (as discussed in the introduction).

The test consists of four trials: 3 × 10 m shuttle run – speed test, standing broad jump – power test, 1 kg medicine ball overhead throw – strength test, 4-min run – endurance test. The results of the individual fitness trials were converted into points (on a scale from 1 to 100), taking into account the age and gender of the participants (32). Along with the physical fitness measurements, anthropometric measurements of body height and weight were also taken. Based on these, body build indices were calculated:

- Body Mass Index (BMI): calculated by dividing body weight in kilograms by the square of height in meters;
- Ponderal Index (PI): calculated by dividing body weight in kilograms by the cube of height in meters.

### 2.3 Study design

The research was conducted between 2015 and 2022 as part of the AFA program led by the Polish Athletics Association. The testers were previously trained in the principles of conducting the OSF test – the research was carried out in accordance with the developed guidelines (32). The authors of this paper received written consent from the Association’s authorities to use the results of the study.

The Bioethics Committee of the ABNS in Biala Podlaska approved the study protocol (Resolution no. 3/2023). This study was conducted within the project “Physical fitness and body build parameters of children and adolescents participating in the Athletics for All! program,” funded by John Paul II University in Biala Podlaska (PB/9/2022).

### 2.4 Statistical analysis

Quantitative variables were presented considering the mean (x̄), median, standard deviation (SD), ranges, and 95% confidence intervals. All statistical tests were conducted separately for each gender, with *p*-values ≤0.05 considered statistically significant. The analyses were performed using SPSS v 17.0 (Softonic, Ashburn, VA, USA).

To verify whether there were statistically significant differences in the means between the groups, a one-way analysis of variance (ANOVA) was used. The Levene Test was used to assess homogeneity. When homogeneity was disturbed, the Welch and Brown-Forsythe tests were used. The assumptions of normality for ANOVA were checked. Only after certain conditions were met did it become possible to use this parametric analysis. In all cases, variance homogeneity was violated, so the Games-Howell post-hoc test was applied, as it handles violations of variance homogeneity and unequal group sizes (33). If the results of the ANOVA analysis were statistically significant, the next step was to examine which specific pairs showed significant differences. The effect size was estimated using the Omega-squared measure (fixed effect), which is interpreted as follows: a value of 0.01 indicates a small effect, 0.06 indicates a medium effect, and 0.14 indicates a large effect (34).

## 3 Results

One-way analysis of variance, conducted to test the hypothesis that the implementation of the AFA program promoting physical activity among Polish youth contributes to the improvement in physical fitness and body build parameters, revealed statistically significant differences in the mean values of measurements taken over the course of eight consecutive years (Tables 2, 3). The results of multiple comparisons between the mean values of all examined variables across the years showed significant differences in most of the analyzed comparisons (Tables 4, 5). It was also found that the percentage of total variability in results between the different years of the study across all age groups, assessed using *𝜔*^2^, was small or medium (1–8%).

**Table 2:** The overall physical fitness of girls and boys in the respective years of the study, within each age group.

Age (years)	Girls	Boys
10	Fwelch (7; 578.096) = 9.596; *p* < 0.001; *𝜔*^2^ = 0.01	Fwelch (7; 402.217) = 6.171; *p* < 0.001; *𝜔*^2^ = 0.01
11	Fwelch (7; 1125.977) = 13.644; *p* < 0.001; *𝜔*^2^ = 0.01	Fwelch (7; 950.923) = 9.585; *p* < 0.001; *𝜔*^2^ = 0.01
12	Fwelch (7; 2415.690) = 31.175; *p* < 0.001; *𝜔*^2^ = 0.03	Fwelch (7; 1683.058) = 11.262; *p* < 0.001; *𝜔*^2^ = 0.01
13	Fwelch (7; 1767.787) = 33.114; *p* < 0.001; *𝜔*^2^ = 0.04	Fwelch (7; 1178.739) = 13.281; *p* < 0.001; *𝜔*^2^ = 0.03
14	Fwelch (7; 432.372) = 31.911; *p* < 0.001; *𝜔*^2^ = 0.07	Fwelch (7; 212.597) = 9.814; *p* < 0.001; *𝜔*^2^ = 0.03
15	Fwelch (7; 237.412) = 23.695; *p* < 0.001; *𝜔*^2^ = 0.08	Fwelch (7; 263.497) = 7.639; *p* < 0.001; *𝜔*^2^ = 0.03

**Table 3:** The values of body build indicators for girls and boys in the respective years of the study, within each age group.

Girls		
Age (years)	BMI	PI
10	Fwelch (7; 574.378) = 4.616; *p* < 0.001; *𝜔*^2^ = 0.006	Fwelch (7; 576.336) = 4.780; *p* < 0.001; *𝜔*^2^ = 0.006
11	Fwelch (7; 1121.773) = 6.685; *p* < 0.001; *𝜔*^2^ = 0.006	Fwelch (7; 1180.486) = 9.396; *p* < 0.001; *𝜔*^2^ = 0.008
12	Fwelch (7; 2404.246) = 21.710; *p* < 0.001; *𝜔*^2^ = 0.02	Fwelch (7; 2406.149) = 26.460; *p* < 0.001; *𝜔*^2^ = 0.02
13	Fwelch (7; 1760.785) = 14.541; *p* < 0.001; *𝜔*^2^ = 0.02	Fwelch (7; 1762.008) = 15.516; *p* < 0.001; *𝜔*^2^ = 0.02
14	Fwelch (7; 432.245) = 4.869; *p* < 0.001; *𝜔*^2^ = 0.009	Fwelch (7; 431.492) = 5.303; *p* < 0.001; *𝜔*^2^ = 0.01
15	No statistically significant differences	No statistically significant differences

Boys		
Age (years)	BMI	PI
10	Fwelch (7; 402.277) = 3.659; *p* = 0.001; *𝜔*^2^ = 0.006	Fwelch (7; 402.506) = 3.641; *p* = 0.001; *𝜔*^2^ = 0.006
11	Fwelch (7; 951.124) = 8.218; *p* < 0.001; *𝜔*^2^ = 0.01	Fwelch (7; 1255.236) = 11.579; *p* < 0.001; *𝜔*^2^ = 0.01
12	Fwelch (7; 1673.848) = 4.736; *p* < 0.001; *𝜔*^2^ = 0.005	Fwelch (7; 1677.475) = 6.405; *p* < 0.001; *𝜔*^2^ = 0.007
13	Fwelch (7; 1173.366) = 3.861; *p* < 0.001; *𝜔*^2^ = 0.006	Fwelch (7; 1175.365) = 4.085; *p* < 0.001; *𝜔*^2^ = 0.007
14	No statistically significant differences	No statistically significant differences
15	No statistically significant differences	No statistically significant differences

**Table 4:** The mean values of variables considering age and year of the study (*N* = 31,789)—girls.

10 years	Variable		Year of study implementation								*F*	Multiple comparisons—statistically significant (*p* < 0.05)
			2015^a^	2016^b^	2017^c^	2018^d^	2019^e^	2020^f^	2021^g^	2022^h^		
	*N* = 4,489		525	986	40	875	871	585	381	226		
	$\tilde{x}$ (SD)	BMI kg/m^2^	16.61 (2.28)	16.7 (2.17)	17.38 (3.28)	16.67 (2.15)	16.9 (2.55)	17.21 (2.40)	16.8 (2.50)	16.59 (2.30)	4.616**^S^	a < f; b < f; d < f; h < f
		PI kg/m^3^	11.41 (1.61)	11.53 (1.53)	12.61 (1.90)	11.61 (157)	11.70 (1.75)	11.78 (1.67)	11.60 (1.75)	11.39 (1.58)	4.780**^S^	a < e; a < f; b < f; h < f
		3x10m (sec.)	9.14 (0.88)	9.12 (0.83)	9.82 (1.04)	8.98 (0.77)	9.12 (1.02)	9.16 (0.89)	9.07 (0.84)	8.93 (0.98)	7.667**^S^	a < c; a > d; b < c; b > d; c > d; c > e; c > f; c > g; c > h; d < e; d < f
		Standing broad jump (m)	1.53 (0.23)	1.53 (0.21)	1.35 (0.21)	1.56 (0.21)	1.57 (0.21)	1.55 (0.21)	1.57 (0.20)	1.58 (0.22)	9.941**^S^	a > c; a > e; a < h; b > c; b < d; b < e; b < h; c < d; c < e; c < f; c < g; c < h
		1 kg medicine ball throw (m)	5.43 (1.22)	5.57 (1.18)	4.66 (1.00)	5.75 (1.30)	5.63 (1.29)	5.77 (1.38)	5.60 (1.40)	5.76 (1.54)	7.929**^S^	a > c; a < d; a < f; b > c; b < d; b < f; c > d; c > e; c > f; c > g; c > h
		4-min run (m)	711.77 (114.79)	737.33 (104.01)	687.25 (80.81)	746.45 (111.68)	729.91 (131.85)	718.46 (122.73)	732.82 (122.38)	721.4 (131.76)	6.515**^S^	a < b; a < d; b > c; b > f; c < d; c < g; d > f
		Total score	231.03 (55.24)	238.29 (52.01)	184.98 (52.87)	245.34 (55.07)	242.41 (56.92)	237.09 (55.19)	241.49 (56.14)	243.76 (60.04)	9.596**^S^	a > c; a < d; a < e; b > c; c < d; c < e; c < f; c < g; c < h
11 years	*N* = 7,108		836	1,352	78	1,320	1,239	956	800	527		
	$\tilde{x}$ (SD)	BMI kg/m^2^	16.95 (2.25)	17.00 (2.20)	18.51 (3.19)	17.16 (2.27)	17.29 (2.26)	17.23 (2.45)	17.16 (2.29)	17.03 (2.34)	6.685**^S^	a < c; a < e; b < c; b < e; c > d; c > e; c > f; c > g; c > h
		PI kg/m^3^	11.24 (1.59)	11.30 (1.48)	12.34 (2.06)	11.38 (1.51)	11.56 (1.53)	11.45 (1.66)	11.34 (1.57)	11.21 (1.60)	9.396**^S^	a < c; a < e; b < c; b < e; c > d; c > e; c > f; c > g; c > h; e > g; e > h
		3x10m (sec.)	8.89 (0.78)	8.79 (0.72)	9.09 (0.90)	8.74 (0.75)	8.79 (0.79)	8.79 (0.89)	8.75 (0.78)	8.68 (0.78)	6.200**^S^	a > b; a > d; a > e; a > g; a > h; c > d; c > g; c > h
		Standing broad jump (m)	1.64 (0.21)	1.64 (0.21)	1.51 (0.25)	1.66 (0.21)	1.68 (0.21)	1.69 (0.23)	1.67 (0.21)	1.71 (0.20)	16.265**^S^	a > c; a < d; a < e; a < f; a < g; a < h; b > c; b < e; b < f; b < g; b < h; c < d; c < e; c < f; c < g; c < h; d < h
		1 kg medicine ball throw (m)	6.22 (1.38)	6.46 (1.36)	6.16 (1.33)	6.58 (1.52)	6.55 (1.52)	6.66 (1.61)	6.53 (1.49)	6.64 (1.61)	7.957**^S^	a < b; a < d; a < e; a < f; a < g; a < h; b < f; c < f
		4-min run (m)	760.32 (109.5)	776.32 (107.61)	725.64 (94.04)	780.89 (113.89)	777.93 (112.09)	788.89 (119.41)	769.34 (121.44)	785.52 (115.47)	7.535**^S^	a < b; a < d; a < e; a < f; a < h; b > c; c < d; c < e; c < f; c < g; c < h; f > g
		Total score	228.99 (54.18)	239.09 (54.00)	206.22 (66.21)	242.96 (56.73)	242.92 (55.85)	246.34 (59.06)	241.11 (58.08)	250.24 (54.61)	13.644**^S^	a < b; a < d; a < e; a < f; a < g; a < h; b > c; b < h; c < d; c < e; c < f; c < g; c < h
12 years	*N* = 8,053		805	1,467	259	1,377	1,257	1,132	914	842		
	$\tilde{x}$ (SD)	BMI kg/m^2^	17.61 (2.32)	17.55 (2.32)	19.51 (3.53)	17.84 (2.31)	17.8 (2.43)	17.85 (2.56)	17.66 (2.46)	17.77 (2.30)	21.710**^S^	a < c; b < c; b < d; b < f; c > d; c > e; c > f; c > g; c > h
		PI kg/m^3^	11.19 (1.50)	11.2 (1.46)	12.55 (2.14)	11.4 (1.52)	11.41 (1.57)	11.51 (1.67)	11.31 (1.60)	11.33 (1.53)	26.460**^S^	a < c; a < d; a < e; a < f; b < c; b < d; b < e; b < f; c > d; c > e; c > f; c > g; c > h
		3x10m (sec.)	8.68 (0.73)	8.55 (0.69)	8.88 (0.74)	8.55 (0.75)	8.61 (0.77)	8.66 (0.82)	8.55 (0.76)	8.49 (0.78)	12.113**^S^	a > b; a < c; a > d; a > g; a > h; b < c; b < f; c > d; c > e; c > f; c > g; c > h; d < f; e > h; f > h
		Standing broad jump (m)	1.73 (0.21)	1.74 (0.20)	1.63 (0.73)	1.77 (0.21)	1.74 (0.23)	1.75 (0.23)	1.77 (0.22)	1.79 (0.22)	13.695**^S^	a < d; a < g; a < h; b < h; c < h; e < h; f < h
		1 kg medicine ball throw (m)	6.68 (1.58)	7.19 (1.48)	6.72 (1.46)	7.35 (1.62)	7.18 (1.57)	7.21 (1.69)	7.23 (1.64)	7.45 (1.63)	20.216**^S^	a < b; a < d; a < e; a < f; a < g; a < h; b > c; b < h; c < d; c < e; c < f; c < g; c < h; e < h; f < h
		4-min run (m)	782.43 (114.59)	814.85 (108.06)	730.15 (108.62)	816.78 (117.72)	804.6 (126.58)	806.63 (124.36)	811.45 (133.80)	802.28 (125.03)	22.035**^S^	a < b; a > c; a < d; a < e; a < f; a < g; a < h; b > c; c < d; c < e; c < f; c < g; c < h
		Total score	229.59 (54.88)	246.25 (51.25)	203.64 (64.34)	249.91 (54.97)	241.94 (58.32)	242.02 (58.56)	247.09 (60.06)	250.55 (55.63)	31.175**^S^	a < b; a > c; a < d; a < e; a < f; a < g; a < h; b > c; c < d; c < e; c < f; c < g; c < h; d > e; d > f; e < h; f < h
13 years	*N* = 5,002		334	654	280	694	728	719	856	737		
	$\tilde{x}$ (SD)	BMI kg/m^2^	18.35 (2.25)	18.75 (2.49)	19.79 (3.14)	18.3 (2.27)	18.45 (2.36)	18.26 (2.30)	18.38 (2.49)	18.47 (2.36)	14.541**^S^	a < c; b < c; b > d; b > f; c > d; c > e; c > f; c > g; c > h
		PI kg/m^3^	11.31 (1.38)	11.59 (1.54)	12.33 (1.98)	11.34 (1.41)	11.47 (1.52)	11.37 (1.46)	11.39 (1.55)	11.43 (1.46)	15.516**^S^	a < c; b < c; b > d; c > d; c > e; c > f; c > g; c > h
		3x10m (sec.)	8.38 (0.73)	8.28 (0.64)	8.71 (0.67)	8.25 (0.71)	8.32 (0.75)	8.42 (0.79)	8.37 (0.80)	8.3 (0.79)	13.553**^S^	a < c; b < c; b < f; c > d; c > e; c > f; c > g; c > h; d < f; d < g
		Standing broad jump (m)	1.84 (0.23)	1.85 (0.21)	1.78 (1.16)	1.85 (0.22)	1.86 (0.22)	1.82 (0.24)	1.85 (0.22)	1.87 (0.22)	2.617*^S^	b > f; e > f; f < h
		1 kg medicine ball throw (m)	6.56 (1.59)	7.01 (1.63)	7.35 (1.50)	8.17 (1.72)	7.92 (1.64)	7.75 (1.75)	8.04 (1.81)	8.32 (1.93)	63.240**^M^	a < b; a < c; a < d; a < e; a < f; a < g; a < h; b < c; b < d; b < e; b < f; b < g; b < h; c < d; c < e; c < f; c < g; c < h; d > f; e > h; f < g; f < h
		4-min run (m)	831.05 (120.24)	833.01 (103.93)	758.45 (99.18)	862.42 (130.48)	843.82 (127.92)	836.23 (129.32)	849.78 (135.79)	840.55 (140.11)	20.956**^S^	a > c; a < d; b > c; b < d; c < d; c < e; c < f; c < g; c < h; d > f; d > h
		Total score	231.15 (58.25)	242.88 (49.72)	206.73 (58.02)	258.76 (54.72)	251.94 (56.67)	243.39 (62.64)	251.30 (56.83)	255.11 (54.60)	33.114**^S^	a < b; a > c; a < d; a < e; a < f; a < g; a < h; b > c; b < d; b < e; b < g; b < h; c < d; c < e; c < f; c < g; c < h; d > f; f < h
14 years	*N* = 3,012		84	534	36	482	333	386	588	569		
	$\tilde{x}$ (SD)	BMI kg/m^2^	18.99 (1.90)	19.23 (2.32)	21.03 (3.17)	19.1 (2.27)	18.93 (2.43)	18.97 (2.89)	18.97 (2.45)	18.79 (2.53)	4.869**^S^	a < c; b < c; c > d; c > e; c > f; c > g; c > h
		PI kg/m^3^	11.49 (1.29)	11.66 (1.43)	12.91 (1.84)	11.61 (1.44)	11.52 (1.50)	11.51 (1.77)	11.53 (1.53)	11.41 (1.54)	5.303**^S^	a < c; b < c; c > d; c > e; c > f; c > g; c > h
		3x10m (sec.)	8.02 (0.52)	8.1 (0.57)	9.39 (0.75)	8.04 (0.61)	7.97 (0.71)	8.09 (0.69)	8.16 (0.78)	8.24 (0.78)	23.125** ^S^	a < c; a < h; b < c; b < h; c > d; c > e; c > f; c > g; c > h; d < h; e < g; e < h; f < h
		Standing broad jump (m)	1.92 (0.22)	1.93 (0.21)	1.55 (0.28)	1.95 (0.21)	1.97 (0.21)	1.96 (0.22)	1.93 (0.21)	1.93 (0.22)	19.701**^S^	a > c; b > c; b < e; c < d; c < e; c < f; c < g; c < h
		1 kg medicine ball throw (m)	6.72 (1.54)	7.12 (1.43)	6.98 (1.27)	8.83 (1.89)	9.03 (1.97)	8.88 (2.20)	8.77 (1.87)	8.72 (2.00)	66.309**^M^	a < d; a < e; a < f; a < g; a < h; b < d; b < e; b < f; b < g; b < h; c < d; c < e; c < f; c < g; c < h
		4-min run (m)	838.56 (130.44)	864.19 (113.00)	718.89 (86.19)	882.23 (128.37)	896.17 (129.27)	874.36 (125.48)	876.32 (136.61)	855.18 (149.72)	11.447**^S^	a > c; a < e; b > c; b < e; c < d; c < e; c < f; c < g; c < h; d > h; e > h
		Total score	232.92 (55.02)	241.29 (50.51)	152.11 (49.47)	260.38 (50.36)	269.87 (53.76)	261.37 (54.46)	256.87 (55.39)	249.45 (55.51)	31.911**^M^	a > c; a < d; a < e; a < f; a < g; b > c; b < d; b < e; b < f; b < g; c < d; c < e; c < f; c < g; c < h; d > h; e > g; e > h; f > h
15 years	*N* = 1891		31	162	25	449	262	330	328	304		
	$\tilde{x}$ (SD)	BMI kg/m^2^	19.39 (1.79)	19.4 (2.12)	19.84 (2.86)	19.73 (2.51)	19.35 (1.92)	19.36 (2.54)	19.43 (2.25)	19.21 (2.36)	1.610**^S^	---
		PI kg/m^3^	11.49 (1.00)	11.66 (1.36)	12.02 (1.77)	11.86 (1.59)	11.67 (1.27)	11.63 (1.56)	11.65 (1.39)	11.53 (1.50)	1.797**^S^	---
		3x10m (sec.)	8.02 (0.55)	8.03 (0.66)	9.78 (1.19)	7.94 (0.66)	7.78 (0.58)	7.94 (0.73)	8.31 (4.00)	8.08 (0.68)	5.661**^S^	a < c; b < c; b > e; c > d; c > e; c > f; c > g; c > h; d > e; e < h
		Standing broad jump (m)	1.95 (0.29)	2.02 (0.20)	1.68 (0.23)	1.99 (0.20)	2.06 (0.21)	2.05 (0.23)	1.98 (0.24)	2.00 (0.24)	14.034**^S^	a > c; b > c; c < d; c < e; c < f; c < g; c < h; d < e; d < f; e > g; e > h; f > g
		1 kg medicine ball throw (m)	7.99 (2.21)	7.56 (1.44)	7.24 (1.26)	9.09 (2.08)	9.51 (2.06)	9.7 (2.10)	9.34 (1.99)	9.53 (2.03)	25.929**^M^	a < e; a < f; a < g; a < h; b < d; b < e; b < f; b < g; b < h; c < d; c < e; c < f; c < g; c < h; d < f
		4-min run (m)	885.74 (101.27)	887.81 (105.00)	741.4 (88.61)	881.82 (120.43)	929.92 (123.95)	905.39 (134.35)	890.17 (141.20)	907.09 (154.70)	8.941**^S^	a > c; b > c; b < e; c < d; c < e; c < f; c < g; c < h; d < e; e > g
		Total score	238.77 (64.63)	244.59 (47.60)	149.4 (43.71)	253.42 (50.99)	275.21 (48.79)	266.7 (54.07)	251.64 (54.60)	257.1 (55.58)	23.695**^M^	a > c; b > c; b < e; b < f; c < d; c < e; c < f; c < g; c < h; d < e; d < f; e > g; e > h; f > g

*The difference in means is significant at the level of 0.05.

**The difference in means is significant at the level of 0.001.

Fixed effect Omega squared: S - 0.01-small effect, A - 0.06-medium effect, L - 0.14-large effect.

**Table 5:** The mean values of variables considering age and year of the study (*N* = 22,260)—boys.

10 years	Variable		Year of study implementation								F	Multiple comparisons – statistically significant (*p* < 0.05)
			2015^a^	2016^b^	2017^c^	2018^d^	2019^e^	2020^f^	2021^g^	2022^h^		
	*N* = 4,489		525	986	40	875	871	585	381	226		
	$\tilde{x}$ (SD)	BMI kg/m^2^	17.19 (2.48)	17.18 (2.29)	18.66 (2.78)	17.48 (2.40)	17.58 (2.39)	17.59 (2.39)	17.44 (2.54)	17.21 (2.39)	3.659**^S^	b < e
		PI kg/m^3^	11.87 (1.82)	11.90 (1.59)	13.11 (1.88)	12.06 (1.72)	12.16 (1.74)	12.08 (1.67)	12.05 (1.81)	11.83*^c^ (1.73)	3.641**^S^	a < c; b < c; h < c
		3x10m (sec.)	9.01 (0.97)	8.81 (0.74)	9.61 (0.87)	8.77 (0.76)	8.87 (0.81)	8.89 (0.94)	8.94 (0.91)	8.82 (0.88)	7.253**^S^	a > b; a < c; a > d; b < c; c > d; c > e; c > f; c > g; c > h
		Standing broad jump (m)	1.59 (0.23)	1.61 (0.21)	1.44 (0.24)	1.62 (0.20)	1.61 (0.20)	1.64 (0.22)	1.61 (0.20)	1.63 (0.19)	4.887**^S^	a > c; a < f; b > c; c < d; c < e; c < f; c < g; c < h
		1 kg medicine ball throw (m)	5.97 (1.22)	6.04 (1.23)	5.32 (1.30)	6.21 (1.38)	6.32 (1.46)	6.35 (1.58)	6.20 (1.47)	6.19 (1.41)	6.117**^S^	a < d; a < e; a < f; b < e; b < f; c < d; c < e; c < f; c < g; c < h
		4-min run (m)	744.87 (142.20)	768.15 (106.05)	776.96 (111.71)	786.07 (120.84)	767.26 (135.46)	769.53 (131.19)	765.95 (124.04)	762.97 (134.74)	4.372**^S^	a < b; a < c
		Total score	227.42 (836)	238.53 (58.68)	194.61 (50.73)	243.05 (62.85)	239.31 (55.29)	241.43 (56.27)	236.74 (57.22)	240.73 (58.83)	6.171**^S^	a < b; a < d; a < e; a < f; b > c; c < d; c < e; c < f; c < g; c < h
11 years	*N* = 5,126		579	1,099	74	945	933	611	551	334		
	$\tilde{x}$ (SD)	BMI kg/m^2^	17.45 (2.27)	17.66 (2.38)	18.57 (3.21)	17.93 (2.61)	18.16 (2.61)	17.98 (2.39)	17.84 (2.66)	17.36 (2.22)	11.579**^S^	a < d; a < e; a < f; b < e; d > h; e > h; f > h
		PI kg/m^3^	11.62 (1.52)	11.76 (1.58)	12.41 (1.79)	12.00 (1.78)	12.16 (1.76)	11.97 (1.68)	11.89 (1.73)	11.46 (1.51)	8.504**^S^	a < c; a < d; a < e; a < f; b < d; b < e; b > h; c > h; d > h; e > h; f > h; g > h
		3x10m (sec.)	8.59 (0.71)	8.52 (0.72)	8.91 (0.75)	8.58 (0.68)	8.70 (0.83)	8.60 (0.86)	8.72 (0.93)	8.48 (0.82)	8.217**^S^	a < c; b < c; b < e; b < g; c > d; c > f; c > h; d < e; d < g; e > h; g > h
		Standing broad jump (m)	1.71 (0.21)	1.71 (0.21)	1.61 (0.22)	1.68 (0.21)	1.69 (0.23)	1.72 (0.22)	1.70 (0.23)	1.76 (0.21)	6.128**^S^	a > c; a < h; b > c; b > d; b < h; c < f; c < g; c < h; d < f; d < h; e < h; g < h
		1 kg medicine ball throw (m)	6.82 (1.58)	6.98 (1.33)	6.27 (1.64)	6.99 (1.60)	7.06 (1.60)	7.14 (1.64)	6.79 (1.57)	7.14 (1.49)	4.793**^S^	a < f; a < h; b > c; c < d; c < e; c < f; c < h; e > g; f > g; g < h
		4-min run (m)	810.18 (123.68)	808.94 (110.37)	775.14 (98.40)	819.54 (125.53)	803.52 (134.08)	828.21 (137.78)	799.05 (135.15)	824.03 (127.07)	9.585**^S^	c < d; c < f; c < h; e < f; f > g
		Total score	235.03 (56.55)	241.03 (54.10)	203.81 (59.53)	237.01 (55.28)	232.20 (59.43)	242.60 (60.39)	228.85 (62.29)	248.56 (59.69)	11.579**^S^	a > c; c < h; b > c; b > e; b > g; c < d; c < e; c < f; c < g; c < h; d < h; e < f; e < h; f > g; g < h
12 years	*N* = 5,328		527	1,012	199	832	881	788	532	557		
	$\tilde{x}$ (SD)	BMI kg/m^2^	18.33 (2.42)	18.00 (2.39)	19.05 (3.41)	18.28 (2.55)	18.38 (2.55)	18.31 (2.54)	18.19 (2.46)	18.20 (2.65)	4.736**^S^	b < c; b < e; c > g; c > h
		PI kg/m^3^	11.65 (1.50)	11.52 (1.51)	12.17 (2.11)	11.68 (1.68)	11.81 (1.66)	11.82 (1.64)	11.58 (1.55)	11.55 (1.65)	6.405**^S^	a < c; b < e; b < f; c > g; c > h
		3x10m (sec.)	8.39 (0.69)	8.31 (0.66)	8.46 (0.69)	8.38 (0.84)	8.45 (0.80)	8.49 (0.89)	8.36 (0.78)	8.33 (0.80)	4.990**^S^	b < e; b < f; f > h
		Standing broad jump (m)	1.81 (0.23)	1.82 (0.22)	1.71 (0.26)	1.82 (0.22)	1.79 (0.24)	1.80 (0.24)	1.81 (0.23)	1.85 (0.24)	10.313**^S^	a > c; a < h; b > c; c < d; c < e; c < f; c < g; c < h; d < h; e < h; f < h
		1 kg Medicine ball throw (m)	7.19 (1.74)	7.76 (1.69)	7.23 (1.54)	7.99 (1.80)	7.97 (1.85)	7.96 (1.84)	8.03 (1.88)	8.05 (1.88)	17.424**^S^	a < b; a < d; a < e; a < f; a < g; a < h; b > c; c < d; c < e; c < f; c < g; c < h
		4-min run (m)	832.52 (138.21)	849.36 (118.65)	793.67 (102.33)	856.41 (131.65)	852.14 (137.51)	840.30 (142.06)	850.79 (138.86)	848.97 (143.08)	6.554**^S^	a > c; a < d; b > c; c < d; c < e; c < f; c < g; c < h
		Total score	227.32 (58.39)	242.37 (55.90)	212.12 (66.43)	244.69 (59.33)	237.77 (63.40)	235.22 (62.78)	242.18 (60.18)	244.66 (62.45)	11.262**^S^	a < b; a < d; a < e; a < g; a < h; b > c; c < d; c < e; c < f; c < g; c < h; d > f
13 years	*N* = 3,278		313	505	167	445	432	433	581	402		
	$\;\;\tilde{x}\;\;\;$ (SD)	BMI kg/m^2^	18.97 (2.60)	18.96 (2.56)	19.82 (3.75)	19.24 (2.45)	18.75 (2.69)	18.83 (2.57)	19.15 (2.84)	18.89 (2.53)	3.861**^S^	c > e; c > f
		PI kg/m^3^	11.43 (1.49)	11.48 (1.44)	12.03 (2.09)	11.66 (1.53)	11.49 (1.61)	11.63 (1.59)	11.76 (1.72)	11.49 (1.55)	4.085**^S^	a < c; b < c
		3x10m (sec.)	7.98 (0.71)	7.96 (0.59)	8.37 (0.81)	8.04 (0.77)	8.07 (0.78)	8.15 (0.77)	8.13 (0.73)	8.02 (0.75)	7.848**^S^	a < c; a < f; b < c; b < f; b < g; c > d; c > e; c > f; c > g; c > h
		Standing broad jump (m)	1.99 (0.24)	1.98 (0.24)	1.83 (0.30)	2.00 (0.27)	1.96 (0.26)	1.91 (0.25)	1.93 (0.23)	1.98 (0.27)	12.713**^S^	a > c; a > f; a > g; b > c; b > f; b > g; c < d; c < e; c < f; c < g; c < h; d > f; d > g; f < h; g < h
		1 kg medicine ball throw (m)	7.58 (1.90)	7.96 (1.89)	8.43 (1.96)	9.36 (2.21)	9.17 (2.13)	8.75 (2.11)	9.08 (2.16)	9.38 (2.04)	40.749**^M^	a < c; a < d; a < e; a < f; a < g; a < h; b < d; b < e; b < f; b < g; b < h; c < d; c < e; c < g; c < h; d > f; f < h
		4-min run (m)	906.84 (127.22)	883.47 (112.18)	807.25 (117.37)	906.53 (150.06)	907.63 (141.76)	897.40 (155.18)	879.52 (150.22)	902.96 (153.84)	11.842**^S^	a > c; b > c; c < d; c < e; c < f; c < g; c < h; e > g
		Total score	235.99 (59.25)	238.12 (58.40)	206.59 (74.14)	254.31 (64.76)	249.04 (63.37)	234.50 (68.20)	238.28 (65.32)	251.42 (64.20)	13.281**^S^	a > c; a < d; a < h; b > c; b < d; b < h; c < d; c < e; c < f; c < g; c < h; d > f; d > g; e > f; f < h; g < h
14 years	*N* = 2,105		55	523	16	317	170	200	427	397		
	$\tilde{x}$ (SD)	BMI kg/m^2^	19.85 (2.72)	19.96 (2.78)	20.84 (2.90)	20.09 (2.35)	19.96 (2.57)	19.70 (2.95)	19.62 (2.84)	19.68 (2.94)	1.473^S^	---
		PI kg/m^3^	11.47 (1.65)	11.58 (1.56)	12.51 (1.96)	11.70 (1.35)	11.58 (1.43)	11.52 (1.67)	11.56 (1.63)	11.54 (1.64)	1.191^S^	---
		3x10m (sec.)	7.78 (0.59)	7.61 (0.53)	8.30 (0.46)	7.65 (0.60)	7.58 (0.73)	7.75 (0.65)	7.88 (0.82)	7.83 (0.85)	9.326**^S^	a < c; b < c; b < g; b < h; c > d; c > e; c > f; c > g; c > h; d < g; d < h; e < g; e < h
		Standing broad jump (m)	2.11 (0.29)	2.18 (0.26)	2.02 (0.33)	2.18 (0.23)	2.19 (0.26)	2.13 (0.28)	2.10 (0.27)	2.12 (0.27)	6.329**^S^	b > g; b > h; d > g; d > h; e > g; e > h
		1 kg medicine ball throw (m)	8.55 (1.91)	8.84 (1.93)	10.06 (1.66)	10.89 (2.28)	10.86 (2.36)	10.73 (2.33)	10.59 (2.38)	10.69 (2.45)	43.977**^M^	a < d; a < e; a < f; a < g; a < h; b < d; b < e; b < f; b < g; b < h
		4-min run (m)	934.89 (148.37)	943.16 (122.20)	888.44 (103.68)	967.51 (144.80)	974.31 (138.42)	945.23 (135.27)	941.68 (172.20)	936.38 (161.41)	2.550*^S^	---
		Total score	214.09 (70.51)	230.11 (59.87)	203.00 (57.07)	256.66 (59.32)	258.99 (64.32)	240.64 (64.78)	232.22 (71.60)	235.57 (66.20)	9.814**^S^	a < d; a < e; b < d; a < e; c < d; c < e; d > g; d > h; e > g; e > h
15 years	*N* = 1,379		25	147	0	361	192	207	208	239		
	$\tilde{x}$ (SD)	BMI kg/m^2^	21.16 (3.58)	21.11 (3.03)	-	20.49 (2.84)	20.62 (2.44)	20.53 (2.86)	20.56 (2.93)	20.51 (2.66)	1.146^S^	---
		PI kg/m^3^	12.10 (1.98)	11.94 (1.71)	-	11.63 (1.64)	11.71 (1.39)	11.57 (1.57)	11.68 (1.70)	11.72 (1.48)	1.166^S^	---
		3x10m (sec.)	7.70 (0.64)	7.52 (0.60)	-	7.49 (0.54)	7.37 (0.66)	7.34 (0.57)	7.73 (1.00)	7.53 (0.65)	7.294**^S^	d > f; d < g; e < g; f < g; f < h
		Standing broad jump (m)	2.20 (0.22)	2.34 (0.24)	-	2.27 (0.22)	2.27 (0.24)	2.32 (0.26)	2.23 (0.28)	2.23 (0.24)	6.036**^S^	b > g; b > h; f > g; f > h
		1 kg medicine ball throw (m)	9.45 (2.14)	10.18 (2.04)	-	11.37 (2.38)	11.96 (2.38)	12.57 (2.31)	12.03 (2.97)	12.41 (2.66)	22.035**^M^	a < d; a < e; a < f; a < g; a < h; b < d; b < e; b < f; b < g; b < h; d < f; d < h
		4-min run (m)	970.00 (119.35)	1001.24 (136.07)	-	986.85 (132.24)	1013.32 (141.56)	1007.43 (148.99)	996.04 (172.66)	964.29 (160.82)	2.720*^S^	e > h
		Total score	207.00 (54.66)	240.54 (53.63)	-	248.09 (56.41)	259.98 (62.92)	265.99 (49.05)	240.50 (68.05)	243.07 (64.11)	7.639**^S^	a < d; a < e; a < f; b < e; b < f; d < f; e > g; f > g; f > h

*The difference in means is significant at the level of 0.05.

**The difference in means is significant at the level of 0.001.

Fixed effect Omega squared: S - 0.01-small effect, A - 0.06-medium effect, L - 0.14-large effect.

A detailed analysis showed that the overall physical fitness of girls (sum of points from individual trials) differed significantly across all age groups at the level of *p* < 0.001. The results of the one-way analysis of variance are presented in Table 2.

The results of the multiple comparisons conducted using the Games-Howell post-hoc test revealed that the overall fitness level in the following years of the study, for both boys and girls and in all age groups, did not show a clear directional trend. However, a clear increasing trend was observed in the following trials: standing broad jump and 3 × 10 m shuttle run for 10-year-old girls, in all trials for 11- and 12-year-old girls, medicine ball throw for 13-year-old girls, standing broad jump, medicine ball throw, and 4-min run for 14-year-old girls, as well as in all trials for 15-year-old girls. In the case of boys, clear increasing trends for all trials were only observed in 10-year-olds. In the remaining age groups, increasing fitness levels were noted for the following trials: standing broad jump, medicine ball throw, and 4-min run for 11- and 12-year-olds, as well as medicine ball throw for 13-, 14-, and 15-year-olds.

The values of body build indicators in the respective years of the study, within each age group and for both genders, differed significantly at the level of *p* ≤ 0.001. In the female groups, unlike the male groups, heterogeneity of variance was observed. The effect sizes from the ANOVA (*𝜔*^2^ < 0.01) indicate that the observed variability in BMI and PI in the studied groups is due to factors other than the year of the study. It is worth noting that the average BMI values in the respective age groups, when referenced to the latest centile charts for the Polish population of children and adolescents (35), oscillate around the 50th percentile. This suggests that the studied youth exhibit a normal, or even exemplary, body build. The results of the one-way analysis of variance for the BMI and PI indicators are presented in Table 3.

The results of the multiple comparisons regarding changes in body build indicators over the subsequent years of the study in most age groups, for both boys and girls, did not show a clear directional trend. Only in the case of 10-year-olds of both genders were positive changes observed for both indicators.

Tables 4, 5 present the average values of the weight-height indicators and the results of individual physical fitness test trials (OSF), taking into account the age of the participants, separately for each gender. The tables show the variability of BMI and PI as well as the PF results over the analyzed eight years. The highest values of the weight-height indicators were recorded in 2017; however, it was the least numerous group.

The conducted analysis of variance revealed numerous statistically significant differences in the compared means (*p* < 0.001). Statistically significant differentiation of results at *p* < 0.05 was observed in the group of thirteen-year-old girls in the standing broad jump trial (the best result was recorded in the last year of the study), as well as in the group of fourteen- and fifteen-year-old boys in the endurance trial. Tables 4, 5 also present the results of multiple comparisons to check if there are differences in the means between more than two study groups (measurements in different years). Statistically significant differences were not found only in the weight-height indicators (BMI and PI) among the two groups of boys (14 and 15 years old).

**Figure 1:**
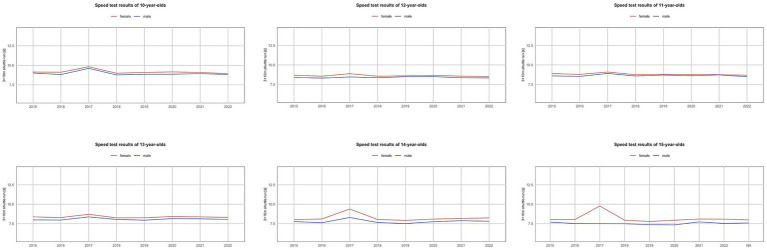
Trends in the speed test results of Polish youth between 2015 and 2022.

**Figure 2:**
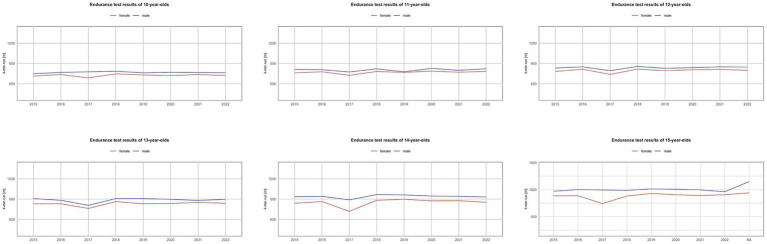
Trends in the endurance test results of Polish youth between 2015 and 2022.

**Figure 3:**
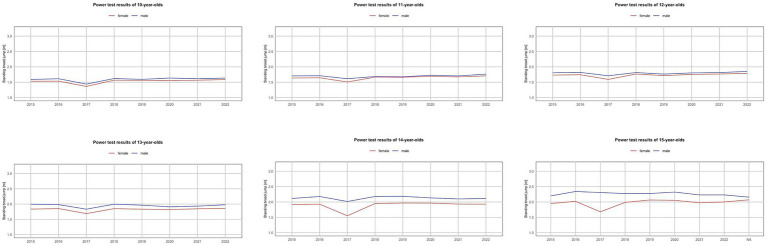
Trends in the power test results of Polish youth between 2015 and 2022.

**Figure 4:**
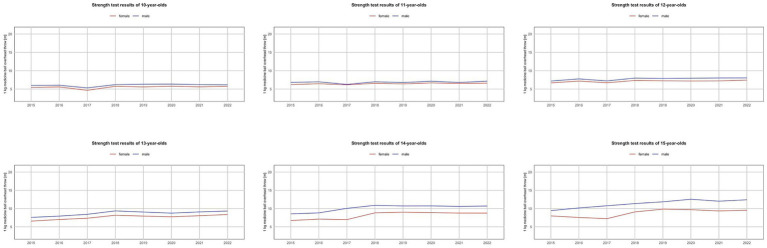
Trends in the strength test results of Polish youth between 2015 and 2022.

Figures 1-4 illustrate trends in the physical fitness of children and adolescents participating in the AFA program. The speed of girls and boys over the eight-year observation period (2015–2022) in individual age groups shows a general tendency to maintain or improve the running time (Figure 1). However, in 2017, a significant decrease in speed was recorded in all examined groups. A favorable trend was also observed in endurance, especially among girls. In the case of boys, a gradual improvement was noted, with the best results in 2018–2019, followed by a decline in endurance. An exception was the group of 11-year-olds, where the best results were achieved in 2020 (Figure 2).

In the power test (measured by the standing long jump distance), an improvement in results was observed between the first measurement in 2015 and the measurement in 2022. The explosive power of the lower limbs of participants in the AFA program showed improvement or relative stabilization over the years 2015–2022 (Figure 3). As for strength (measured by the medicine ball throw distance), progress was recorded in both girls and boys (Figure 4), with a high statistically significant variation (Tables 4, 5).

**Figure 5:**
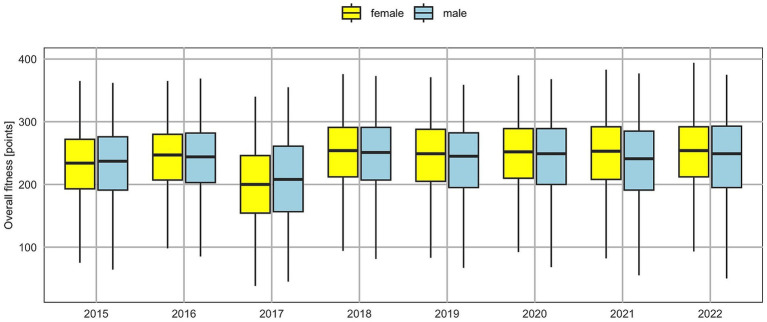
Physical fitness trends of Polish youth.

The results presented in Figure 5 (individual test results converted into points according to the OSF methodology) showed that over the eight analyzed years, participants of the AFA program of both genders demonstrated a high level of PF, i.e., they became stronger, faster, more enduring, and exhibited greater explosive lower-body power. The only exception was the test results from 2017.

## 4 Discussion

Physical fitness in children and adolescents is an extremely important asset for their future health (4, 36). However, population studies rarely address secular changes in PF, even though monitoring these trends is crucial for obtaining information necessary for appropriate interventions. The present study aims to illustrate trends in PF among children and adolescents participating in the national AFA program. In Poland, a clear trend of declining physical activity among children and adolescents has been observed. According to the Global Matrix 4.0 report on the state of physical activity in children and adolescents, a decreasing percentage of young people meet the World Health Organization (WHO) recommendations. Such observations are more difficult to confirm regarding sedentary behaviors (including screen time) and how quickly their characteristics change (37). There is strong and consistent evidence of beneficial relationships between overall physical activity (PA) and obesity, several cardiometabolic biomarkers (cholesterol, blood pressure, triglycerides), as well as physical fitness components such as aerobic capacity, muscle strength, and endurance (38). Children and adolescents who do not meet WHO recommendations are likely to suffer from “effort deficit syndrome,” which carries negative health consequences. Due to a sedentary lifestyle, they are more susceptible to adverse health effects in later stages of life (39).

An active lifestyle and established patterns of engaging in physical activity during childhood and adolescence persist into adulthood (40).

Our study provides novel findings on trends in PF among Polish children and adolescents participating in the AFA program. The results of research conducted between 2015 and 2022 showed that their level of physical fitness increased with age. Similarly, a study by Santos et al. (41) conducted among Peruvian children found that both girls and boys demonstrated significantly higher PF levels as they aged. Findings presented by Bahan et al. (42) from research on Irish primary school students indicate that the relationship between fundamental movement skills (FMS) and health-related fitness (HRF) is dynamic and generally strengthens with age during childhood. Children acquire and refine a wide range of fundamental motor skills that enable them to perform more complex movements. They learn to run, jump, catch, and throw, as well as combine various fundamental motor skills, which may contribute to improved PF assessment results. The process of developing fundamental motor skills follows a progression principle, after which internalization occurs, leading to skill refinement. As noted by Santos et al. (41), achieving higher PF levels with age requires systematic, guided exercise under the supervision of a coach or a teacher. Our findings align with this perspective.

Our study revealed a favorable trend in PF levels of Polish children participating in the AFA program over the analyzed years. Considering the average point scores, girls in 2021 achieved higher values than boys. Additionally, no significant changes were observed in other PF tests during the study period. The most significant finding of this study is the improvement in specific PF components in both genders, contrary to the general trends suggesting a decline in PF among children and adolescents in various countries over recent decades. This decline has been associated with the development of unhealthy lifestyles from an early age. It may seem that researchers from Canada (43) reached similarly positive conclusions, reporting that the PF levels of Canadian children and adolescents remained relatively stable. However, the observed lack of improvement in PF, combined with evidence that most Canadian children and adolescents are insufficiently active (with sedentary behaviors prevailing), suggests that efforts to enhance physical fitness and promote healthy, active behaviors among young people have been inadequate. These findings highlight the need for implementing programs aimed at increasing physical activity among children and adolescents, including initiatives such as the AFA program. Ongoing monitoring of PF trends can serve as a valuable tool for planning future interventions to improve physical fitness at the population level.

The study conducted on Polish university students indicates that secular trends in somatic development are not always accompanied by favorable changes in motor fitness. Progress has been more evident in running and jumping trials, while a decline has been observed in throwing performance (44). The positive impact of lifestyle and environmental changes in recent years is reflected in the findings of Costa et al. (45). Similar to our study, these researchers reported improvements in physical fitness results in Portuguese children – except for the standing broad jump. Their study showed that between 2009 and 2013, children performed better in speed and strength tests compared to their peers from 20 years earlier. Other researchers have demonstrated a sharp decline in children’s aerobic capacity since 1970, whereas anaerobic capacity has remained relatively stable in this age group. The authors suggest that this trend may be influenced by social, behavioral, physical, psychosocial, and physiological factors (46).

In a systematic review by Fühner et al. (47) covering the years 1972–2015, the authors observed a significant initial increase followed by an equally large subsequent decline in cardiorespiratory endurance. This decline appeared to reach its lowest point for all children between 2010 and 2015. Researchers from Lithuania (48) and Finland (49), based on measurements conducted over the past two decades (after 2000), reported stagnation in endurance levels among children and adolescents. Relative muscle strength measures showed a general trend of slight increase, with no significant gender-related effects, though the trend was more pronounced in boys. In contrast, muscle power indicators demonstrated a slight overall negative trend. Regarding speed measures, a small to moderate increase has been observed in recent years.

In another systematic review conducted by Masanovic et al. (50), 19 studies analyzing data from 1,746,023 children and adolescents from 14 countries (China, Finland, Sweden, Belgium, New Zealand, Denmark, Spain, Norway, Mozambique, Poland, the USA, Lithuania, Portugal, and Canada) between 1969 and 2017 were included in the analysis. The authors noted that most studies reported a consistent decline in strength and endurance. Findings from studies conducted on the Chinese population indicated an increase in strength between 1985 and 1995, followed by a decline until 2014. Similar patterns were observed in endurance levels. Regarding speed, trends varied depending on the population. Research by Matton et al. (51) suggests a negative trend in speed for both boys and girls.

The results of the study by Karpowicz et al. (52) conducted among young female basketball players confirm the downward trend in physical fitness. This trend is also reflected in the findings of Dong et al. (53), who analyzed data from a group of 12.5 million children between 1985 and 2014. The authors observed an overall decline in physical fitness levels of the participants. Similarly, a systematic review by Eberhard et al. (36), which included 24 studies from 16 countries with a sample of over 860,000 children and adolescents, reported a general decline in physical fitness levels in most of the analyzed studies.

Wilczewski and Wilczewski (54), who studied secular trends in the motor fitness of school-aged boys from the central-eastern region of Poland between 1986 and 2016, highlight an increased rate of body mass gain. As a result, there has been a rise in BMI as well as an increase in the percentage of children and adolescents with overweight and obesity. The researchers note that this excessive weight gain relative to height, combined with the widely observed decline in physical activity, contributes to a decrease in physical fitness of children and adolescents (54–56). Similarly, the study conducted on Portuguese youth has also shown an increasing trend in BMI (45). The findings presented by our research team confirm the necessity of implementing programs that promote physical activity on a broad scale. Participants of the AFA program exhibit exemplary body build. The average BMI values of the study participants, when referenced against percentile charts (35), fluctuate around the 50th percentile.

### 4.1 Strengths and limitations

Physical fitness was measured using a validated and standardized tool – the OSF test – which has the significant advantage of being easy to administer. This means that data collection was based on objective and reliable PF tests, conducted according to detailed (rigorous) guidelines. In our analysis, we considered gender, age, and BMI/PI as key factors influencing PF. However, we acknowledge that other variables, such as socioeconomic status or athletic experience (regular participation in sports activities), also affect PF trends. Notably, strong evidence exists linking physical fitness levels with biological maturity (57). Research by Bellis et al. (58) has indicated a trend toward earlier maturation over the past few centuries. This has been accompanied by an increase in physical fitness, which may have influenced the observed trends (the age of puberty onset was not accounted for in our study).

The nationwide scope and large sample size represent clear strengths of the presented research findings.

The year 2017 was an exception to the other years covered by the analysis. The sample size in that year was by far the smallest, amounting to 1,229 people (out of a total of 54,049 respondents). This was because the AFA program did not require the OSF test to be performed. Instead, the Ministry of Sport introduced a requirement to perform the International Physical Fitness Test. Only some coaches decided to perform additional measurements using the OSF test. The limited scope of the study (sample size) may be one of the reasons for the unusual results recorded during this period.

The studies did not include formal adjustments for confounding factors, and the absence of a control group limits the ability to clearly attribute the observed effects to the AFA program. Furthermore, the study did not include formal adjustments for confounding factors, and the absence of a control group limits the ability to clearly attribute the observed effects to the AFA program. Therefore, the results should be interpreted with caution.

Future studies plan to include such a control group, which would allow for a more complete assessment of the impact of the AFA program on physical fitness development. Additionally, it is worth considering an analysis that takes into account variables such as the biological age of participants and their length of participation in the program, which would allow for more objective and comparable results.

## 5 Conclusion

This study provides up-to-date information on the physical fitness of Polish children and adolescents. The results indicate that physical fitness levels have remained relatively stable over nearly a decade, which, in an era dominated by a sedentary lifestyle among youth, confirms the validity of implementing physical activity promotion programs (including the AFA program). Differences in PF trends based on gender and age are minimal. It is essential to emphasize that assessing physical fitness is crucial for monitoring trends and predicting future interventions aimed at improving it at the population level. Regular annual implementation of physical fitness tests can play a key role in identifying children and adolescents with low physical fitness, ultimately contributing to the promotion of positive health behaviors.

In the context of the presented research results, it is extremely important to focus on promoting the participation of children and adolescents in extracurricular physical activities. The ‘Athletics for All’ program, aimed at this age group, provides an opportunity for regular participation in sports activities, and through regular testing, it allows for self-monitoring of motor skills – the progress in the development of the key components of physical fitness, also in a health context. It is also important to note that in Poland, there is no uniform strategy for determining physical fitness levels (as highlighted by the authors of the report within the Global Matrix 4.0 project). Therefore, considering the scope of the AFA program, we confirm the validity of its implementation. Furthermore, we hope that the results presented by our team will contribute to the development of effective public policies.

## Data Availability

The raw data supporting the conclusions of this article will be made available by the authors, without undue reservation.

## Footnotes

1 Available online at: www.lekkoatletykadlakazdego.pl (Accessed 10 November 2024).
