# Supplementary material for: Trends in physical fitness among polish children and adolescents
Source: Front Public Health. 2025 Nov 26;13:1576822. doi: 10.3389/fpubh.2025.1576822 (PMC12689887; doi:10.3389/fpubh.2025.1576822)
Supplement: Supplementary file 1 [file Table_1.DOCX]

**FLOW CHART OF PARTICIPANTS IN THE STUDY**

Excluded (n=3,979)

- the lack of a reference group (n=900)
- insufficient program participation time (n=2,346)
- the small sample sizes of certain age groups (733)

n=747 (400 F and 347 M) 8-year-olds;

n=153 (83 F and 70 M) 17-year-olds

n=54,049 young individuals

(31,789 F and 22,260 M)

**Identification**

n=2,346 (1,332 F and 1,014 M) 9-year-olds

n=733 (419 F and 314 M) 16-year-olds

participation in the AFA program

written consent of a parent or legal guardian

**The inclusion criteria**

age range of 8 to 17 years

n=50,070 participants of the AFA program

**Included**
